# Identification of the collagen type 1 alpha 1 gene (*COL1A1*) as a candidate survival-related factor associated with hepatocellular carcinoma

**DOI:** 10.1186/1471-2407-14-108

**Published:** 2014-02-19

**Authors:** Masamichi Hayashi, Shuji Nomoto, Mitsuhiro Hishida, Yoshikuni Inokawa, Mitsuro Kanda, Yukiyasu Okamura, Yoko Nishikawa, Chie Tanaka, Daisuke Kobayashi, Suguru Yamada, Goro Nakayama, Tsutomu Fujii, Hiroyuki Sugimoto, Masahiko Koike, Michitaka Fujiwara, Shin Takeda, Yasuhiro Kodera

**Affiliations:** 1 Gastroenterological Surgery (Department of Surgery II), Graduate School of Medicine, Nagoya University, 65 Tsurumai-cho, Showa-ku, Nagoya 466-8550, Japan

**Keywords:** Hepatocellular carcinoma, Collagen type 1 alpha 1, Methylation

## Abstract

**Background:**

Hepatocellular carcinoma (HCC) is one of the major causes of cancer-related death especially among Asian and African populations. It is urgent that we identify carcinogenesis-related genes to establish an innovative treatment strategy for this disease.

**Methods:**

Triple-combination array analysis was performed using one pair each of HCC and noncancerous liver samples from a 68-year-old woman. This analysis consists of expression array, single nucleotide polymorphism array and methylation array. The gene encoding collagen type 1 alpha 1 (*COL1A1*) was identified and verified using HCC cell lines and 48 tissues from patients with primary HCC.

**Results:**

Expression array revealed that *COL1A1* gene expression was markedly decreased in tumor tissues (log_2_ ratio –1.1). The single nucleotide polymorphism array showed no chromosomal deletion in the locus of *COL1A1*. Importantly, the methylation value in the tumor tissue was higher (0.557) than that of the adjacent liver tissue (0.008). We verified that expression of this gene was suppressed by promoter methylation. Reactivation of *COL1A1* expression by 5-aza-2′-deoxycytidine treatment was seen in HCC cell lines, and sequence analysis identified methylated CpG sites in the *COL1A1* promoter region. Among 48 pairs of surgical specimens, 13 (27.1%) showed decreased *COL1A1* mRNA expression in tumor sites. Among these 13 cases, 10 had promoter methylation at the tumor site. The log-rank test indicated that mRNA down-regulated tumors were significantly correlated with a poor overall survival rate (*P* = 0.013).

**Conclusions:**

Triple-combination array analysis successfully identified *COL1A1* as a candidate survival-related gene in HCCs. Epigenetic down-regulation of *COL1A1* mRNA expression might have a role as a prognostic biomarker of HCC.

## Background

Liver cancer is the fifth most common cancer in men and the seventh in women [1]. Each year, hepatocellular carcinoma (HCC) is diagnosed in more than half a million people worldwide [2]. Liver resection is the treatment of choice for HCC. However, recurrence is observed in 77–100% of the patients within 5 years of the surgery [3]. The 5-year survival rate remains poor, at around 50% [4], indicating that intensive postoperative management is required. In general, we have some options for postoperative treatment, including local radiofrequency ablation (RFA), transarterial chemoembolization (TAE), radioembolization, and molecular targeted therapy. Establishment of more precise prognostic determinants using molecular biology techniques is warranted to make the best use of these options. In the current study, surgical samples and matched clinical data were used to identify a prognostic marker, focusing on the genomic alterations of hepatic carcinogenesis.

We combined gene expression array analysis and single nucleotide polymorphism (SNP) array analysis to gain whole genome information. The gene expression profile provides a snapshot of the transcriptional state of noncancerous and tumor tissues. SNP array is a useful tool for surveying the loss of heterozygosity (LOH), a prominent characteristic of many human cancers. We combined the use of these two arrays in one representative surgical sample and found several tumor-specific gene alterations [5-10] (Table 1).

**Table 1:** **Information of genes detected by double or triple**-**combination array analysis**

**Gene symbol**	**Function of encoded protein**	**Expression in tumor (log2 ratio)**	**SNP array**	**Methylation value in tumor and noncancerous liver**	**Methylation in HCC cell lines**
*MT1G*	A preserver of biologically essential metals homeostasis	-3.1~3.6	No LOH	N/A	3/5 (60.0%)
*EFEMP1*	A family of extracellular matrix protien	-3.7~4.1	No LOH	N/A	7/9 (77.8%)
*LIFR*	A component of signaling complex in IL-6 cytokine family	-3.7~5.1	No LOH	N/A	6/6 (100.0%)
*FBLN1*	A family of extracellular matrix protein	-2.8~3.4	No LOH	N/A	4/5 (80.0%)
*RELN*	A family of extracellular matrix protein	-3.0	No LOH	N/A	2/5 (40.0%)
*AKAP12*	A scafford protein of protein kinase A signaling pathway	-2.8	No LOH	N/A	3/6 (50.0%)
*BLMH*	A cytoplasmic cysteine peptidase	-1.3	No LOH	0.530 / 0.089	2/5 (40.0%)
*ESR1*	A nuclear hormone receptor	-2.5	No LOH	0.775 / 0.093	5/9 (55.6%)
*DCDC2*	An enhancer of microtubule polymerization	-2.2	No LOH	0.846 / 0.212	6/9 (66.7%)
*DNM3*	A member of dynamin family and related to endocytosis	-1.0	No LOH	0.879 / 0.213	8/9 (88.9%)
*COL1A1*	A major component of type I collagen	-1.1	No LOH	0.557 / 0.084	4/6 (66.7%)

LOH, loss of heterozygosity; N/A, not applicable.

HCC is known as one of the human cancer types in which methylated promoter CpG islands are frequently found [11]. We therefore added methylation array results of the same HCC samples to complete the triple-combination array method, which is designed to search for epigenetic alterations more efficiently. This method has already succeeded in identifying potentially useful candidate prognostic markers [12-15] (Table 1). The aim of this study was to identify further hitherto unknown tumor-related and survival predictive genes in HCCs using data from the same arrays.

In this study, we decided to use the collagen type 1 α1 (*COL1A1*) gene as a tumor-related gene from the results of the triple-combination arrays. This human gene encodes the α1 chain of type I collagen, the major extracellular matrix (ECM) component of skin and bone. More than 90% of patients with osteogenesis imperfecta have abnormalities in *COL1A1* or *COL1A2*[16]. Type I collagen has also been reported to be one of the components of hepatic fibrosis [17]. Because no study had revealed the correlation of *COL1A1* with HCC, we aimed to evaluate the relevance of *COL1A1* expression in HCC samples.

## Methods

### Sample collection

In 2007, partial hepatectomy was performed in a 68-year-old woman (hereafter referred to as the “study patient”) who was found to have a 3-cm HCC derived from chronic hepatitis C. Specimens were immediately excised from both the tumor tissue and the adjacent noncancerous liver tissue.

Six HCC cell lines (Hep3B, HLE, HLF, HuH2, HuH7, SK-Hep1) were obtained from the American Type Culture Collection (Manassas, VA, USA). The cell lines were cultured in RPMI-1640 medium (Invitrogen, Carlsbad, CA, USA) supplemented with 10% fetal bovine serum and incubated in 5% CO_2_ at 37°C.

A total of 48 tumor tissues and adjacent noncancerous liver tissues were collected from patients who had undergone hepatectomy and had been diagnosed as having primary HCC tumors at Nagoya University Hospital during 1994–2001. Written informed consent, as required by the institutional review board, was obtained from all patients. The median follow-up period was 92.7 months (range 18.2–213.1 months).

### Expression array analysis

Expression array analysis was performed using total RNA extracted from the study patient’s tumor tissue and adjacent noncancerous tissue. Total RNA was isolated from each of the frozen samples using an RNeasy Mini Kit (Qiagen, CA, USA) according to the manufacturer’s protocol. Gene-expression profiles were determined using Affymetrix HGU133A and HGU133B GeneChips (Affymetrix, Santa Clara, CA, USA). Double-stranded complementary DNA (cDNA) was synthesized from 8 μg of total RNA with oligo d (T)^24^ T7 primer. Biotinylated cRNA (20 μg) was denatured at 94°C for 35 min and hybridized to a human Genome U133 Plus 2.0 GeneChip array (Affymetrix). The hybridized cRNA probes were processed for signal values using Micro Array Suite 5.0 software (Affymetrix).

### SNP chip array analysis

The SNP chip array experiments were also conducted using the study patient’s tumor and noncancerous tissue according to the standard protocol for GeneChip Mapping 500 K arrays (Affymetrix). Total genomic DNA was digested, ligated, and subjected to a polymerase chain reaction (PCR) using a single primer. PCR products were labeled with a biotinylated nucleotide analogue and hybridized to the microarray. Hybridized probes were captured by streptavidin–phycoerythrin conjugates, and the array was scanned and genotypes identified. All copy number analyses were performed using the Copy Number Analyzer for Affymetrix GeneChip Mapping 500 K arrays (CNAG) version 2.0.

### Methylation array analysis

Methylation array analysis was conducted using the study patient’s tumor and noncancerous tissue according to the standard protocol for Illumina Infinium HumanMethylation27 Beadchip Kit (Illumina, San Diego, CA, USA). Genomic DNA (1 μg) was bisulfite-converted using the EpiTect Bisulfite Kit (Qiagen) in accordance with the manufacturer’s instructions. Bisulfite-converted DNA was hybridized to the HumanMethylation27 BeadChip. Methylation levels of each CpG site were determined with fluorescent signals for methylated and unmethylated alleles.

### RT-PCR analysis

Total RNA (10 μg) was isolated from 6 HCC cell lines, 48 primary HCC tissues, and corresponding noncancerous liver tissue. These samples were used to generate complementary DNA (cDNA). The cDNA was amplified by PCR primers for *COL1A1* sense (S) strands (5′-TCTGCGACAACGGCAAGGTG-3′ in exon2) and antisense (AS) strands (5′-GACGCCGGTGGTTTCTTGGT-3′ in exon3), which amplified a 146-base pair (bp) product. After the initial denaturation step (94°C for 5 min), reverse transcription (RT)-PCR amplification was undertaken, consisting of 30 cycles of 94°C for 12 s, 60°C for 8 s, and 72°C for 8 s. RT-PCR of β-actin was also performed to confirm the amounts of cDNA for each sample. PCR products were loaded directly onto 3% agarose gels, stained with ethidium bromide, and visualized under ultraviolet illumination.

### Real-time quantitative RT-PCR analysis

The PCR reactions were performed with the SYBR Green PCR Core Reagents Kit (Applied Biosystems, Foster City, CA, USA) under the following conditions: 1 cycle at 95°C for 10 s and then 40 cycles at 95°C for 5 s and at 60°C for 30 s. Real-time detection of the SYBR Green emission intensity was conducted with an ABI prism 7000 Sequence Detector (Applied Biosystems). The primer pairs used for RT-PCR were also used here. For standardization, expression of glyceraldehyde-3-phosphate dehydrogenase (*GAPDH*) (TaqMan; Applied Biosystems) was quantified for each sample [18]. The *COL1A1* gene expression level was defined as the value obtained from real-time quantitative RT-PCR analysis divided by the *GAPDH* value.

### Methylation-specific PCR

For DNA methylation analysis, 2 μg of genomic DNA was subjected to sodium bisulfite conversion of unmethylated cytosines using the EpiTect Bisulfite Kit (Qiagen) in accordance with the manufacturer’s instructions. The primer pairs for methylated detection were specific to the *COL1A1* promoter region: S (5′-TTGGTTGGGGTACGGGCGGT-3′) and AS (5′-CCTCACACTCCGCGTACCTC-3′), which amplify a 154-bp product. In contrast, those for unmethylated detection were specific to the same region: S (5′-GATTGGTTGGGGTATGGGTG-3′) and AS (5′-CCTCCTACTCCAACCCCAAA-3′), which amplify a 140-bp product. The methylation-specific PCR (MSP) amplification consisted of 40 cycles at 94°C for 12 s, 60°C for 8 s, and 72°C for 8 s. The unmethylation-specific PCR (UMSP) consisted of 40 cycles at 94°C for 12 s, 58°C for 8 s, and 72°C for 8 s after the initial denaturation step (94°C for 5 min).

### 5-Aza-2′-deoxycytidine treatment

To confirm that promoter methylation had led to silencing of gene expression, six HCC cell lines were treated with a DNA methylation inhibitor, 5-aza-2’-deoxycytidine (5-aza-dC) (Sigma-Aldrich, St. Louis, MO, USA). Cells were seeded at a density of 1.5 × 10^6^/ml on day 0. The medium with 5-aza-dC (10 μM) was changed on days 1, 3, and 5. After incubation, cells were harvested on day 6, and the RNA was extracted. RT-PCR was performed as described above.

### Sequence analysis

Genomic bisulfite-treated DNAs from HCC cell lines were sequenced. PCR was conducted in the *COL1A1* promoter region for the sequencing. The PCR primer pairs were S (5′-GGGTAGGGTTTTTTTTTGTTTTT-3′) and AS (5′-CTAAACCCTAAACATATAAACTC-3′), which amplify a 179-bp product. PCR amplification consisted of 35 cycles of 94°C for 15 s, 51°C for 12 s, and 72°C for 12 s after the initial denaturation step (94°C for 5 min). PCR products were purified directly using the QIAquick PCR Purification Kit (Qiagen). Finally, purified templates were prepared for direct sequencing using the BigDye Terminator version 1.1 Cycle Sequencing Kit (Applied Biosystems) and the BigDye Xterminator (Applied Biosystems). Sequence analysis was carried out using an Applied Biosystems ABI310, and sequence electropherograms were generated using ABI Sequence Analysis software version 5.1.1.

### Western blotting analysis

Cultured cells were washed and lysed by Pierce RIPA buffer (Thermo Fisher Scientific, Madison, WI, USA). Protein lysates were homogenized and then underwent centrifugation. The supernatant was used for the analysis. The protein concentration was calculated using the Pierce BCA Protein Assay Kit (Takara Bio, Ohtsu, Japan). NuPAGE LDS sample buffer (Invitrogen) was added to each adjusted protein sample and resolved on 10% sodium dodecyl sulfate polyacrylamide gel. Electrotransfer was performed to polyvinylidene fluoride membranes using the iBlot Gel Transfer Device (Invitrogen) and blocked in 5% nonfat dry milk. Membranes were immunoblotted overnight at 4°C with a mouse anti-COL1A1 antibody (SAB1402151; Sigma–Aldrich, St. Louis, MO) followed by peroxidase-conjugated secondary antibodies. For β-actin, a mouse monoclonal anti-β-actin antibody (Abcam, Cambridge, UK) was used. Signals were detected by enhanced chemiluminescence (Lumivision PRO HSII, Aisin Seiki, Kariya, Japan).

### Immunohistochemical staining

Sections were treated with 3% H_2_O_2_ to inhibit endogenous peroxidase and were then subjected to antigen retrieval using 10 mM citrate buffer at 95°C for 10 min, repeated five times. Sections were incubated with Histofine SAB-PO (R) (Nichirei, Tokyo, Japan) for 10 min and with a mouse anti-COL1A1 antibody (SAB1402151; Sigma Aldrich) diluted 1:1000 in ChemMatet antibody diluent (Dako, Copenhagen, Denmark) overnight. EnVision (Dako) was used as a secondary antibody. Staining was developed for 3 min using liquid diaminobenzidine as the substrate (Nichirei). We determined staining properties using vessels as an internal control.

### Statistical analysis

Continuous variables were compared using the Mann-Whitney U-test. Categorical variables were compared using the χ^2^ test or Fisher’s exact test, where appropriate. Overall survival rates were analyzed by the Kaplan-Meier and log-rank tests. All statistical analyses were performed using JMP 9 software (SAS institute, Cary, NC, USA). The level of statistical significance was set at *P* < 0.05.

## Results

### Triple-combination array

**Figure 1:**
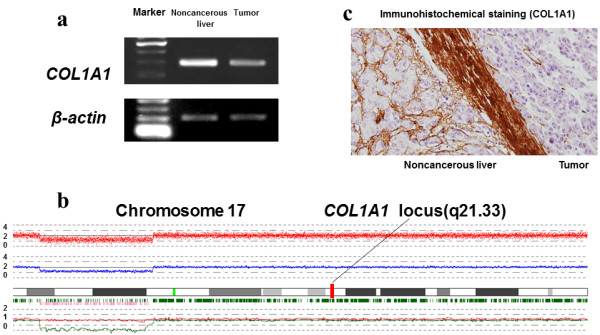
**Primary data for surgical samples from a 68**-**year**-**old woman (study patient). a**. Down-regulation of the *COL1A1* gene was seen in the tumor tissue compared with the adjacent noncancerous liver tissue (146 bp). Reverse transcriptase polymerase chain reaction (RT-PCR) for β-actin was performed to normalize the quantity of cDNA. **b** Copy number analysis of chromosome 17. There was no deletion or amplification at the *COL1A1* gene locus (17q21.33). **c** Immunohistochemical staining of COL1A1 protein showed that tumor tissue components showed almost no staining compared with adjacent noncancerous tissue components (200×).

We first searched for candidate tumor suppressor genes by expression array analysis, focusing on genes with more decreased expression levels in HCC tissue than adjacent noncancerous tissue. Consequently, *COL1A1* was found to show decreased expression at a level of −1.1 in the log 2 ratio (Table 2a). Then, SNP array was conducted for the same samples. Chromosomal deletions were observed at 3q, 8p, 11q, 12q, 16p, 17p, 19p, and X. Chromosomal gains were observed at 1q, 3q, 11q, 12p, and 12q. There were no copy number abnormalities recorded in chromosome 17q, where *COL1A1* is located (Figure 1b). One of the SNP signals showed a heterozygous AB allele in both the normal and tumor samples (Table 2b). These results suggested that *COL1A1* expression was diminished without chromosomal deletion. We then checked the methylation array data for the same samples (Table 2c). The methylation value (0–1.0) of the tumor tissue was significantly higher (0.557) than that of the adjacent noncancerous liver tissue (0.084). As a result, we hypothesized that decreased expression of *COL1A1* gene in tumor tissue was influenced by promoter methylation.

**Table 2:** **Results of triple**-**combination array of a 68**-**year**-**old woman**’**s** (**study patient**) **surgical samples a expression array analysis of *COLIAI***

**a** Expression array analysis of *COL1A1*								
Probe set ID	Gene symbol	Log2 ratio	Noncancerous liver signal	Detection	Tumor signal	Detection	Probe ID	Chromosomal location
1556499_s_at	*COL1A1*	-1.1	2171.4	P	912.1	P	HU133p2_03053	chr17q21.33
202310_s_at	*COL1A1*	-1.1	493.4	P	193.2	P	HU133p2_11759	chr17q21.33
**b** Single-nucleotide polymorphism (SNP) signals of *COL1A1* gene locus								
Probe set ID	Chromosome	Physical position	Noncancerous liver		Confidence	Tumor		Confidence
SNP_A-2189880	17	13542410	AB			0.265625	AB		0.046875
SNP_A-420153	17	13542446	AA			0.000488	AA		0.028320
SNP_A-2200119	17	13548602	BB			0.007813	BB		0.007813
**c** Methylation array analysis of *COL1A1*									
Probe ID	Gene symbol	Sample	Methylation value	Status			Confidence	Chromosomal location	
				Total	Methylated	Unmethylated			
cg01593886	*COL1A1*	Noncancerous liver	0.084	11893	1010	10883	3.678E-38	chr17q21.33	
		Tumor	0.557	7512	4240	3272	3.678E-38		

### “Study patient” samples and HCC cell lines

**Figure 2:**
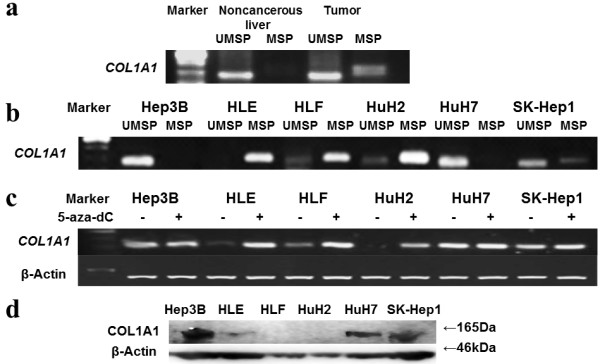
**Analysis of *COL1A1 *methylation and expression. a** Promoter methylation status of the study patient’s samples was examined. Methylation-specific PCR (MSP) and unmethylation-specific PCR (UMSP) were performed. Only tumor tissue had promoter methylation. **b** Promoter methylation status of the *COL1A1* gene six hepatcellular carcinoma (HCC) cell lines. Complete methylation was detected in the cell line HLE; partial methylation in HLF, HuH2, and SK-Hep1; and complete unmethylation in Hep3B and HuH7. **c***COL1A1* expression was reactivated in HLE, HLF, HuH2, and SK-Hep1 by 5-aza-2′-deoxycytidine (5-aza-dC) treatment. β-Actin was used as the normalization gene. **d** COL1A1 protein expression was confirmed by western blotting. Very weak or no band was detected in the cell lines with positive promoter methylation. β-Actin was used as the normalization gene.

To verify our hypothesis, we first confirmed that *COL1A1* mRNA expression and COL1A1 protein were decreased in the study patient’s tumor tissue (Figure 1a, c). Above all, *COL1A1* promoter methylation in the tumor tissue was confirmed (Figure 2a).

**Figure 3:**
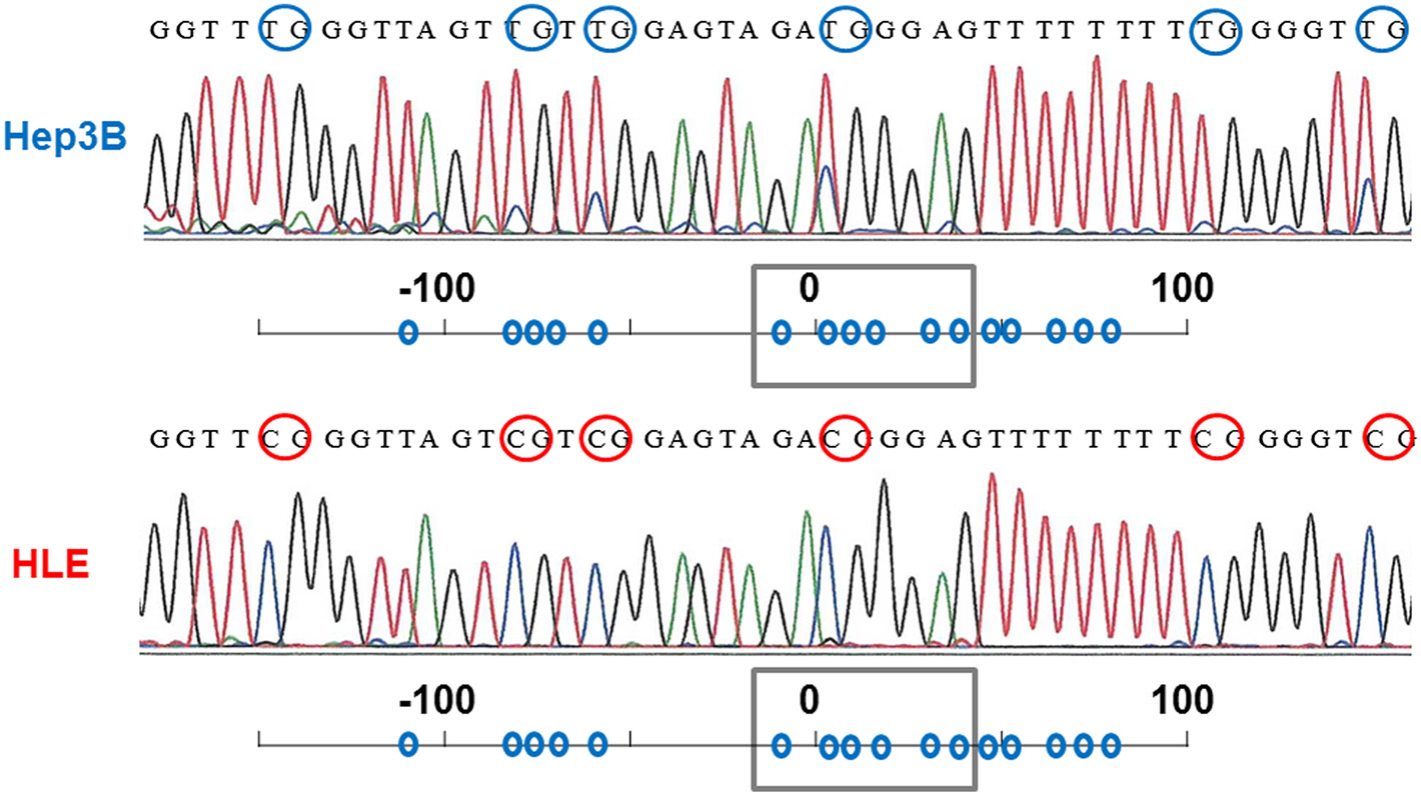
**Direct sequencing of bisulfite**-**treated HCC cell lines.** The location was between −12 and +35 bp from the transcription initiation site. All CG dinucleotides were almost unmethylated (blue circles) in Hep3B. In contrast, all CG dinucleotides were completely methylated (red circles) in HLE.

We also conducted both MSP and UMSP in six HCC cell lines (Figure 2b). We subsequently identified almost complete methylation in HLE cells; partial methylation in HLF, HuH2, and SK-Hep1 cells; and no methylation in Hep3B or HuH7 cells. To confirm that amplifications of both PCRs were correctly performed, bisulfite sequencing was examined [19]. CpG dinucleotides of Hep3B were almost unmethylated, and those of HLE were all methylated (Figure 3). These results verified the accuracy of MSP and UMSP.

**Figure 4:**
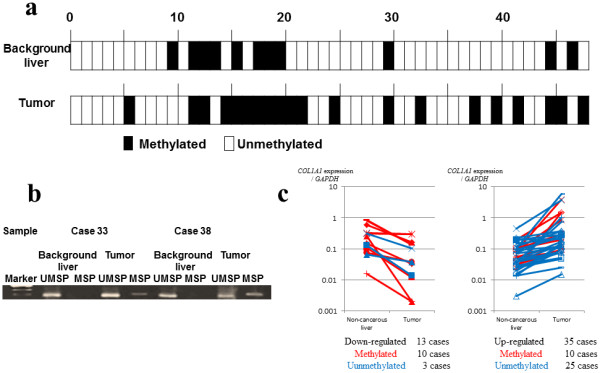
**Promoter methylation status in 48 tumor tissues and matched noncancerous liver tissues. a** A total of 20 tumor tissues and 11 noncancerous tissues showed promoter methylation. **b** MSP and UMSP results of two representative cases. **c** The 48 cases were divided into13 *COL1A1* expression down-regulated cases and 35 up-regulated cases in tumor tissues. Promoter methylated cases were indicated by red lines. Promoter methylation and the mRNA expression pattern were significantly correlated (*P* = 0.002).

We next examined whether promoter methylation led to the silencing of *COL1A1* gene expression by treatment with 5-aza-dC, a DNA methylation inhibitor. After 5-aza-dC treatment, the methylated cells showed reactivation of *COL1A1* mRNA expression (Figure 2c). Concerning the expression of COL1A1 proteins by western blotting analysis, unmethylated cell lines showed high-intensity bands, whereas mainly methylated cell lines showed weak or no bands (Figure 4). The results were consistent with the MSP and UMSP results.

### Surgical samples of 48 HCC patients

We then aimed to evaluate the *COL1A1* promoter methylation status in 48 surgical samples. Among the 48 tumor tissues, 20 (41.7%) showed *COL1A1* promoter methylation (Figure 4a). Among these 20 methylated cases, 8 also showed methylation in noncancerous tissues. MSP and UMSP results of two representative cases are shown in Figure 4b. From the viewpoint of mRNA expression, 10 of 13 down-regulated cases had promoter methylation in tumor tissues, whereas 25 of 35 up-regulated cases had no methylation in tumor tissues (Figure 4c). Significant correlation was found between down-regulation of mRNA expression and tumor methylation (*P* = 0.002).

**Figure 5:**
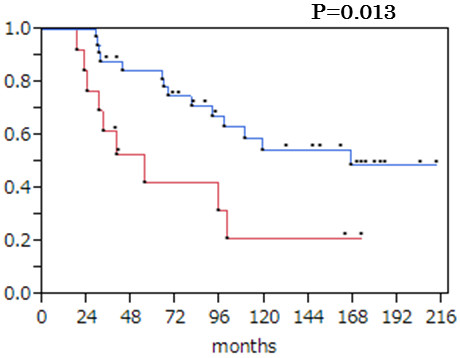
**Overall survival curves for down**-**regulated and up**-**regulated cases of *COL1A1 *mRNA expression in tumor tissues.** Red line: down-regulated (n = 13). Blue line: up-regulated (n = 35). According to the log-rank test, down-regulated cases were significantly correlated with poor overall survival (*P* = 0.013).

Finally, we analyzed the correlation between *COL1A1* mRNA expression and clinicopathological features of the 48 HCC patients (Table 3). Down-regulated cases were significantly correlated with worse liver damage scores (*P* = 0.011) and capsule formation (*P* = 0.026), both of which are correlated with background liver fibrosis [20] and methylation in the tumor (*P* = 0.002). The down-regulation also correlated (log-rank test) with poor overall survival rate (*P* = 0.013) (Figure 5). In the multivariate analysis, only liver damage and liver cirrhosis were significant factors for overall survival (data not shown).

**Table 3:** **Correlation between *COL1A1 *** **mRNA expression and clinicopathological features**

**Variable**	**Definition**	**Down-regulated (n=13)**	**Up-regulated (n=35)**	**P**
Age	≥65 years / <65 years	7 / 6	16 / 19	0.620
Sex	female / male	3 / 10	2 / 33	0.100
Virus	HCV / HBV / none	11 / 2 / 0	27 / 5 / 3	0.374
Child classification	B / A	2 / 11	3 / 32	0.507
Liver damage score	B / A	9 / 4	10 / 25	0.011
Tumor size	≥50 mm / <50 mm	3 / 10	10 / 25	0.700
Tumor number	multiple / solitary	4 / 9	13 / 22	0.685
Differentiation	poor / mod / well	0 / 12 / 1	1 / 30 / 4	0.699
Growth type	invasive / expansive	1 / 12	4 / 29	0.654
Capsule formation	present / absent	13 / 0	27 / 7	0.026
Septum formation	present / absent	10 / 2	26 / 7	0.732
Serosal invasion	present / absent	0 / 7	4 / 16	0.105
Vessel invasion	present / absent	1 / 12	9 / 26	0.176
Cirrhosis	present / absent	9 / 4	14 / 21	0.077
AFP	≥20 ng/ml / <20 ng/ml	10 / 3	18 / 17	0.115
Japanese stage	III + IV / I + II	4 / 9	12 / 23	0.820
TNM stage	III + IV / I + II	2 / 11	8 / 27	0.562
Methylation in tumor	present / absent	10 / 3	10 / 25	0.002

HCV, hepatitis type C virus; HBV, hepatitis type B virus; AFP, alpha-fetoprotein.

## Discussion

Collagen is one of the most characteristic substances seen in liver fibrosis. Especially, collagen type IV is available as a marker of hepatitis C fibrosis [21]. Collagen types I, II, and III have also been reported to be associated with the liver fibrosis stage of chronic HCV [22]. Koilan et al. [17] showed that the end-product of fibrosis is abnormal synthesis and accumulation of type I collagen in the ECM, which is produced by activated stellate or Ito cells in the damaged liver. Our data also support this idea because *COL1A1* gene expression levels of pathologically cirrhotic 25 noncancerous liver tissues are significantly higher than those in 23 noncirrhotic liver tissues (*P* = 0.010). In addition, Kao et al. showed hepatoma-derived growth factor (HDGF), which was correlated with the progression of HCC, also stimulated the production of collagen type 1 [23]. HDGF overexpression promoted the synthesis of TGF-β1 and COL1A1, leading to enhanced collagenous matrix deposition in liver. Lin et al. also reported that *COL1A1* expression was usually up-regulated in invasive HCC [24]. This might be why *COL1A1* expression in the tumor is usually higher than that of adjacent noncancerous liver tissue.

On the other hand, epigenetic alterations of collagen genes have been reported in various neoplasms. Collagen type I is composed of three polypeptide chains transcribed from two separate genes, *COL1A1* and *COL1A2*. Each gene is methylated in several human cancer cells with coordinately decreased collagen expression [25]. Concerning the *COL1A1* gene, frequent promoter methylation was detected in renal cell carcinoma [19], and decreased expression was found in ovarian serous carcinoma [26]. *COL1A2* gene expression was epigenetically down-regulated in medulloblastoma [27], melanoma [28,29], head and neck cancer [30], and bladder cancer [31].

Taken together, as for our 48 samples, although *COL1A1* mRNA is usually up-regulated in tumor tissues, there is a small group of tumors that has down-regulated mRNA expression mainly due to promoter methylation. Those down-regulated cases were correlated with poor overall survival. All patients received no adjuvant chemotherapy. During the follow-up period of each patient, 9 out of 13 down-regulated cases and 19 out of 35 up-regulated cases had recurrences. None of the former recurrent cases received any treatment, whereas 10 of the latter recurrent cases received surgery (3 cases) or TAE (6 cases) or RFA (1 cases). Although the difference might influence the survival data of each group, some untreatable reasons, like multiple liver metastasis, distant metastasis or sever hepatic dysfunction, might be correlated with recurrences in *COL1A1* down-regulated cases. In connection with this result, Dahlman et al. [32] reported that there was a tendency toward a negative correlation between the ability to produce collagen type I and tumorigenicity in the xenograft mouse model of anaplastic thyroid cancer cell lines. This is because collagen type I-producing cancer cells separate themselves from surrounding stromal components that are essential for tumor growth. Conversely, collagen type I-lacking cancer cells might easily come into contact with stromal components. These two entities may stimulate each other, resulting in cancer progression. Indeed, suppression of ECM metalloproteinase was proved to lead to inhibition of cell growth and migration [33]. This result means that the ECM of tumor cells, which consists mainly of collagen type I, functions to block tumor cells from spreading. Moreover, Zeller et al. [34] identified *COL1A1* as one of the methylated genes in cisplatin-resistant ovarian cancer cells, which is usually related to poor clinical outcomes [35]. The acquisition of drug resistance results from repopulation of the tumor with inherently drug-resistant cancer-sustaining cells [36]. *COL1A1* gene methylation might be correlated with the poor prognostic characteristics of cancer-sustaining cells.

Recently, cancer therapy targeting epigenetic alterations has emerged [37,38]. The promising targets are DNA methyltransferases and histone deacetylases, which are being studied in a number of ongoing clinical trials. Combined therapy with these two drugs appears to be a rational strategy for anticancer treatment [39]. However, epigenetic therapy is generally less effective in solid tumors than in hematological malignancies because solid tumor carcinogenesis usually consists of multiple genomic alteration steps. Above all, it is difficult for epigenetic therapies to target only the specific gene locus.

Huang et al. reported on micro RNA-152 regulated DNA methyltransferase 1 (*DNMT1*) mRNA expression in hepatitis B-related HCCs [40]. *DNMT1* is one of the methylation controller genes that maintain the methylation pattern in the newly synthesized DNA strand for epigenetic inheritance. Another report indicated that there is some cross-talk between epigenetics and micro-RNAs in hepatocarcinogenesis [41]. Micro-RNA might therefore be a convenient tool for regulating the methylation status of target epigenetic alterations. As we have a well-established method for detecting cancer-related methylated genes, searching the correlation between micro-RNA expression and epigenetic alterations might be the next strategy for understanding hepatocarcinogenesis.

One of the problems with our results was that the methylation occurred not only in tumor tissues but also in some noncancerous liver tissues. When both samples were methylated, consistent down-regulation of the *COL1A1* mRNA in the tumor was not observed. This was why several mRNA up-regulated cases were found in tumor-methylated cases (Figure 5). The log-rank test revealed that methylated cases of noncancerous liver were associated with poor recurrence-free survival (*P* = 0.031) and poor overall survival (*P* = 0.044). In addition, most methylated cases of noncancerous tissues also had methylation in the tumor tissues. Thus, it is possible that a certain precarcinogenic status is already established in methylated noncancerous samples. To confirm this finding, we must examine the methylation status of completely normal liver tissues in a future study.

## Conclusions

Our triple-combination array analysis facilitated the search for yet unknown tumor-related genes in HCC. Although a significant correlation was not indicated in the multivariate analysis of this small cohort, epigenetic down-regulation of *COL1A1* mRNA expression in tumor tissues might be a candidate prognostic factor of HCC.

## Abbreviations

cDNA: Complementary DNA; COL1A1: Collagen type 1 alpha 1; ECM: extracellular matrix; HCC: Hepatocellular carcinoma; HDGF: hepatoma-derived growth factor; LOH: Loss of heterozygosity; MSP: Methylation-specific PCR; PCR: Polymerase chain reaction; Radiofrequency ablation: RFA; SNP: Single nucleotide polymorphism; Transarterial chemoembolization: TAE; UMSP: Unmethylation-specific PCR.

## Competing interests

The authors declare that they have no competing interests.

## Authors’ contributions

MH: data acquisition and drafting of the manuscript; SN: study concept and design, data acquisition, and study supervision; MH, YI, MK, YO, and YN: data acquisition; CT, DK, SY, GN, TF, HS, MK, MF, ST, and YK: samples collection and critical review of the manuscript. All authors approved the final manuscript.

## Pre-publication history

The pre-publication history for this paper can be accessed here:

http://www.biomedcentral.com/1471-2407/14/108/prepub
